# Book Reviews

**Published:** 1862-10

**Authors:** 


### OUR LIBRARY TABLE.

*On Mechanical Appliances in the Treatment of Deformities.* By HENRY HEATHER BIGG, Assoc. Inst. C.E. London: John Churchill. 1862.

This volume offers to the profession a complete exposition of all that is known and approved of on the subjects of which it treats. Mr. Bigg has for many years had the largest opportunities which extensive practice in London, and diligent research in continental schools afford, of maturing and fashioning into a scientific system, that branch of operative surgery he has specially adopted. It is not too much to say that he has eminently succeeded in so doing. His work deserves to rank amongst the best professional treatises of the day, worthy to be placed as a text-book by the side of Cooper or Liston. In the age of rude surgery, mechanical appliances as a means of remedying deformity were but little understood; as the science of anatomy in its relation to physiology unfolded the mysteries of vital mechanics, crude notions of the treatment of malformations congenital or acquired, by degrees gave way to a more enlightened system. The mechanician for scientific purposes assumed the position of the operating surgeon. The result was, as might have been anticipated, cases formerly regarded as hopeless, were brought within the category of those which admitted of cure, and affections which, though not considered as beyond remedy, yet were ever sources of prolonged suffering, became divested of all that was formidable, and required but competent and timely interference for their comparatively easy, and with rare exceptions, certain removal. Mr. Bigg's work abounds with evidence of this character, and cannot fail to impress its readers with a high view of its author's exertions in the particular branch of science to which it is devoted. Many most valuable instruments and methods for the removal of deformities are fully detailed, and freely offered to the profession. Instruments which if patented, could not fail to prove a source of considerable emolument to the author, their inventor, are clearly demonstrated. Mr. Bigg takes a higher view of his profession than that of a pecuniary or personal one; and we cannot do better than quote his own words on this particular point.

"Often as it has been stated, that to publish a description of those inventions resulting from long study and great opportunities, must eventually lead to such diffusion as shall react against the interests of those who originally promulgate their value; it can never be a matter of pride or satisfaction to any one to feel that his inventive faculties are exercised solely for the sordid motive of personal emolument. It is far nobler to offer to the world all the knowledge and information on a special subject which one possesses, than to hoard its advantages, and be the only one to profit by its employment. There is also a certain reward secured to those who freely proffer their plans and designs, by the fame attached to them for the possession of ingenuity of thought and integrity of action. Holding these views, I freely volunteer such knowledge and experience as I may possess, and place them at the disposal, not only of those who need their aid, but also of my own *confrères*, simply begging them to be guided by the same rule when opportunity offers."

For the readers of this Journal, Mr. Bigg's work has special attraction. The connexion between mind and body is so intimate and mysterious, the abstruse and close sympathies which exist, reflecting the obliquities of the one in the malformations of the other, bring under the notice of the Psychologist perhaps more than other practitioners, illustrations of those several deformities Mr. Bigg suggests and lays down remedies for. We believe that the merits of this work will secure its introduction into the library of every asylum and public institution throughout the kingdom. It is written in a practical, truthful, and honest spirit, and as such we recommend its perusal to the profession and public.

*Consumption, its Early and Remediable Stages.* By Edward Smith, M.D., LL.B., F.R.S., Assistant-Physician to the Hospital of Consumption and Diseases of the Chest, &c. London, 1862, pp. 44.

This is one of those books which are the despair of the reviewer. It so abounds in details that a quotation of its table of contents would alone suffice to give an accurate notion of its scope and character. In the preface the author states, that in writing the work he had four principal objects in view, viz., "to take advantage of the growing belief of the day that there is a stage of phthisis in which the disease is as remediable as it is irremediable at a later period; to write a practical work in which may be faithfully represented the actual condition of those cases when regarded in the great number in which they have been brought before his observation; to treat the subject as far as possible, on the inductive method, and on the improved physiology and pathology of the day; and to give practical effect to numerous series of special inquiries which have been made by him during the preceding seven years."

On the first object of the author we offer the following citation from his preliminary remarks :—

"Tubercle is almost universally regarded as the essential feature of the disease, the cause of its progress in the lungs, and the source of injury to the general system; and the great desideratum has been its removal. The general system has been regarded in two aspects; as associated with the deposition of tubercle, and as influenced by the deposition; and the evil has not been in regarding it in this double light, but in giving an undue prominence to the latter, when considering the relation of tubercle to phthisis. In truth, modern views have so engrossed the mind, that one condition in the progress of phthisis has (because it is always found at some period) been raised from the minor to the dignity of the major premiss; and instead of the statement that all cases of tubercle (in the lungs) are phthisis, it is averred that in all cases of phthisis there is tubercle.

"We venture to affirm that this consideration is worthy of careful attention in any attempt to obtain a true view of the nature of this disease.

"If tubercle be due to pre-existent causes, whatever may be their nature, the disease is not in the tubercle, but in the cause of the tubercle; and the tubercle itself is but a result—an evidence, and all the changes in the tubercle are secondary influences. It cannot be affirmed that the tubercular element of the disease possesses an innate power of destruction such as is found in the elements of cancer, for whatever may be the origin of tubercle in the lungs, it is found upon free surfaces, and does not spread by extension, whilst cancer infiltrates the tissues and has the power of extension, and consequently of displacement or destruction. Tubercle itself is a foreign but a passive agent; and all actions, of whatever kind, which proceed about it originate and are carried on, not by the tubercle, but by the containing tissues. It is placed in the lungs and accumulates by new deposits; and the subsequent changes are not due to any disintegrating action in the tubercular element, but in the tissues which contain it.

"Hence surely the tubercle is not the essence of the disease, but only one of the results—a result doubtless met with at some, but not at every period in the progress of each case. So long as inquirers fix their attention upon this or any other single product of the disease, they will fail to recognise its true nature; and so long as the aim of the practical man is to find a plan whereby the tubercle may be absorbed or rejected, he also will fail. We therefore think that whilst our age has made the great advance of distinguishing more carefully those cases of wasting which are associated with a particular disease of the lungs from those due to other causes, and has therefore given to us a large group of cases of a similar character, it has greatly erred in fixing its attention upon one of the conditions of the lungs, and regarding it, not as one of many results of diseased action, but as the essence of the disease, and the prime cause of mischief. It has cast aside that minute attention to the general system which was the especial subject of observation of the fathers of medicine, and has been content to concentrate all its powers of observation upon one internal condition which has this in common with the external symptoms, viz., that both are effects of the disease, and not the disease itself. We have limited our cases to one class only, and have spent our time in determining the characteristics of that class, but have made little progress in that higher department of knowledge—the determination of the true nature of the disease."

On the second and third objects of the author we may say that they have been most faithfully achieved; and of the third object, it may be said, that, in carrying it out, the high estimate we formed of the practical value of the author's researches, when reviewing his work on "Cyclical Changes in Health and Disease," has been most fully confirmed. The present work, indeed, is a boon to the working practitioner. In it he will find whatever is known of the nature and evidences of the early stages of phthisis, and whatever light a wide experience and most elaborate series of researches directed to the subject has thrown upon the rationale and system of treatment, as well as valuable hints on climate and data on prognosis.

The following extract relative to *food*, taken from the "categorical statement of the whole plan of treatment," and which is a summary of details which have previously been discussed at length, will suffice to illustrate the practical character of this valuable book:—

"The patient should take from two to three pints of milk daily, prepared (and we also add, *thickened*) with chocolate, arrowroot, flour, gluten semola, oatmeal, or bread; or made with eggs, &c., into puddings. In cases where new milk does not agree, skimmed milk may be in part supplied, and then, if fats be tolerated, half an ounce of suet, cut finely, should be well boiled in each pint of milk, and taken quite warm. The milk should be eaten in somewhat small quantities, say half a pint at a time; one quantity is to be taken immediately on the patient awaking in the morning, others at breakfast and supper, the milk pudding for dinner, and chocolate or coffee may be added to the milk which is taken at breakfast and tea. Food should further be taken at intervals of from two to three hours, and the dinner should be supplied soon after mid-day. Half a pint of good soup, with bread, may be taken between breakfast and dinner, and, if fats are not disliked, it would be better to prepare the soup from ox-heads or shins, so as to supply both oil and jelly in addition to the juices of the meat; and the whole should be well thickened with groats, or corn flour. Eggs, bacon, or meat, should be taken at breakfast, and an abundance of fresh meat at dinner, with soup, pudding, and a moderate quantity of fresh vegetables, French beans, and bread. The meat should be of the richest quality, and have at least one-third of its weight of fat. If the patient like salad oil, it may be eaten as freely as possible. A small quantity of cheese should be added to the dinner. An egg should be taken at the tea meal, and also at supper when milk is not taken; there should also be a cup of milk and bread-and-butter placed at the bedside of the patient, and eaten, if possible, during the night. Beer, or wine, may be taken at dinner, and once or twice at other periods of the day if it be found to agree with the system, and the dose be so moderate that it may not in the least affect the head, or cause heaviness in, and indisposition to move, the limbs. Usually, wine should be taken with hot water; but when the progress of the case is satisfactory, alcohols are not necessary. All food should be taken hot, and prepared so as to please the taste of the patient."

*The Spas of Europe.* By JULIUS ALTHAUS, M.D., M.R.C.P. London, 1862. pp. 494.

This work will be a most welcome addition to the library of the physician. In it Dr. Althaus supplies us with a comprehensive description of the physical properties, chemical composition, geographical distribution, physiological action, and therapeutical uses of mineral water. To announce so important and useful a work, coming from the pen of a competent authority, is alone necessary to ensure its success. A want has long been felt in our literature, for a work of ready reference on the different characteristics of the numerous spas scattered over the surface of Europe, which may be made available for the treatment of disease. This work Dr. Althaus has now happily supplied.

When Dr. Lodwick Rowzee published his little book on "The Queene's Welles—that is, A Treatise on the nature and virtues of *Tunbridge* Water, (1632) Dr. Argent, the then President of the College of Physicians, and Ottwell Meverell and Richard Spicer, Fellows of the said College, prefaced the treatise, according to the custom of the times, with the following remarks:—

"As diverse medicinable waters are daily found out in many places, so is it a very profitable labour to make true observation of their effects and best manner of using them, especially by men of learning and judicious understanding, and such as have been accustomed to the *frequent use* of them, both *in themselves* and others, whereby they may make their observations more true and certaine. Such a one wee take this Author to be, concerning the Waters neere *Tunbridge*, whose paines taken herein wee doubt not but will be very usefull to all such as shall have occasion to make tryall of them."

Now that the College of Physicians is reviving so many of its privileges and customs long forgotten or laid aside, it is to be hoped that if it should ever again have recourse to approval of the literary productions of its members, it will eliminate from the reasons of such approval, the frequent personal use of medicaments, whether naturally or artificially concocted. It is lamentable to contemplate the state of an authority on European Spas, whose opinion was to be received as of weight, only so far as he had made use of the different waters discovered, in his own person. For the rest, however, the present President and Fellows of the College might with some modification adapt the approval of Dr. Rowzee's book in favour of Dr. Althaus.

*Neuerahr: a New Spa on the Rhine.* By JAMES MILLER, F.R.S.E., Professor of Surgery in the University of Edinburgh. Edinburgh, 1861. pp. 35.

A delightful account of a new alkaline acidulous spa, which we commend to all who are sent annually spa-ward by rheumatism and gout.

*Psychological Inquiries. 2nd Part. Being a Series of Essays intended to illustrate some points in the Physical and Moral History of Man.* By Sir Benjamin C. Brodie, Bart., D.C.L., F.R.S. London, 1862. pp. 247.

The second series of these charming dialogues needs but a brief notice on publication at the hands of the reviewer. The importance of self-knowledge, the influence of external circumstances on the condition of the mind, human happiness, the advantage to be derived from the intercourse of different classes of society with each other, and natural theology, are some of the subjects touched upon in the present volume. It is to be regretted, however, that a remark of Baron Alderson, addressed to a jury, should be quoted with approval:—" The prisoner (the learned Judge observed) is said to have laboured under an uncontrollable impulse to commit the crime; the answer to which is, that the law has an equally uncontrollable impulse to punish him." The averment was an uncontrollable impulse arising from *diseased mind;* and the proposition might, to adopt Baron Alderson's play upon words, be said equally to apply to the action of the law upon the subject. For, although the law may not recognise a morbid impulse of this character, it does not follow that such an impulse did not exist in the case which was in question before the judge, or that the assumption of its existence was not consistent with fact and observation. There was simply question of its non-recognition by the law. But it is most certain that such impulses directly arising from disease, and attended by the most serious consequences, have occurred and do occur, and that it is a mockery to assert that an individual affected by an uncontrollable impulse of this nature would be morally responsible, whatever the legal responsibility might be. It is not for medicine to confine its teachings within the strait limits required, for very obvious purposes, by the law. It is for medicine to see that these limits are not so drawn as to exclude cases which reason, experience, and observation alike teach us are provided for by the spirit, whatever the letter of the law may be.

*On Ovarian Dropsy: its Nature, Diagnosis, and Treatment: the Result of Thirty Years' Experience.* By J. Baker Brown, F.R.C.S., Senior Surgeon to the London Surgical Home for Diseases of Women. 8vo. London, 1862. pp. 283.

In this work Mr. Baker Brown has brought together the results of his long and extensive experience of ovarian dropsy. He has instituted an impartial examination of the comparative merits of the different methods of treatment advised, added a practical account, with cases, of the operation for extirpating the whole tumour, and he has endeavoured to show in what cases and under what circumstances this formidable interference is justifiable. The work will command the earnest attention of every practical surgeon.

*Defects in the Treatment of Insanity in the Public Lunatic Asylums of Ireland, with suggestions for their Remedy, and some Observations on English Asylums.* By John A. Blake, M.P. 8vo, 1862, pp. 103.

Mr. Blake brought the subject of this pamphlet before the House of Commons in the course of the past Session of Parliament. We postpone the consideration of his examination of this important question until a subsequent number.

On our table we have also Dr. Edward Smith's elaborate paper on the *Elimination of Urea and Urinary Water, in relation to the Period of the Day, Season, Exertion, Food, Discipline, Weight of Body, and other influences acting in the Cycle of a Year*, from the last Volume of the Philosophical Transactions; and among reprints and new editions, Dr. Townley's *Parturition without Pain;* Dr. Cutler's *Notes on Spa* (5th edition); Dr. M'Intosh's *Notes on Asylums and the Insane in France and Belgium* (July and August, 1861), a most valuable paper: also by the same gentleman a paper *On the Subcutaneous Injection of Morphia in Insanity;* Dr. Laycock on *The Antagonism of Law and Medicine in Insanity, and its Consequences*, the important Introductory Lecture given before the Class of Medical Psychology at the University of Edinburgh, in the late Session; Dr. Yellowlee's singularly interesting history of a case of *Homicidal Mania;* and *Our Moral Relations to the Animal Kingdom*, a digest of the statements of the Bible in respect thereto.

We have also before us a paper from the Asylum for Idiots, Earlswood, Red-hill, Surrey, containing a copy of the Report of the Commissioners in Lunacy on the state of the Asylum, in June last. It is gratifying to find that the Commissioners speak in the highest terms of this most admirable institution, the welfare of which we commend to the favourable consideration of our readers.

